# Diagnostic accuracy of lung ultrasound in diagnosis of ARDS and identification of focal or non-focal ARDS subphenotypes: a systematic review and meta-analysis

**DOI:** 10.1186/s13054-024-04985-1

**Published:** 2024-07-08

**Authors:** Maud M. A. Boumans, William Aerts, Luigi Pisani, Lieuwe D. J. Bos, Marry R. Smit, Pieter R. Tuinman

**Affiliations:** 1 Department of Intensive Care Medicine, Noordwest Ziekenhuisgroep, Wilhelminalaan 12, Alkmaar, The Netherlands; 2 Department of Intensive Care Medicine, Amsterdam UMC, Vrije Universiteit Amsterdam, De Boelelaan 1117, Amsterdam, The Netherlands; 3 Department of Intensive Care Medicine, Amsterdam UMC, Amsterdam Medisch Centrum, Meibergdreef 9, Amsterdam, The Netherlands; 4 Mahidol-Oxford Tropical Medicine Research Unit (MORU), Mahidol University, Bangkok, 10400 Thailand; 5 Amsterdam Leiden IC Focused Echography (ALIFE), Amsterdam, The Netherlands; 6 Amsterdam Cardiovascular Sciences, Amsterdam UMC, Vrije Universiteit Amsterdam, De Boelelaan 1117, Amsterdam, The Netherlands; 7 Amsterdam Institute for Immunity and Infectious Diseases, Amsterdam UMC, Vrije Universiteit Amsterdam, De Boelelaan 1117, Amsterdam, The Netherlands; 8 Department of Precision-Regenerative Medicine and Jonic Area (DiMePRe-J), Section of Anesthesiology and Intensive Care Medicine, University of Bari “Aldo Moro”, Bari, Italy; 9 Laboratory of Experimental Intensive Care and Anesthesiology (LEICA), University of Amsterdam, Meibergdreef 9, Amsterdam, The Netherlands

**Keywords:** ARDS, Lung ultrasound, Respiratory medicine, Diagnostic test accuracy

## Abstract

**Background:**

Acute respiratory distress syndrome (ARDS) is a life-threatening respiratory condition with high mortality rates, accounting for 10% of all intensive care unit admissions. Lung ultrasound (LUS) as diagnostic tool for acute respiratory failure has garnered widespread recognition and was recently incorporated into the updated definitions of ARDS. This raised the hypothesis that LUS is a reliable method for diagnosing ARDS.

**Objectives:**

We aimed to establish the accuracy of LUS for ARDS diagnosis and classification of focal versus non-focal ARDS subphenotypes.

**Methods:**

This systematic review and meta-analysis used a systematic search strategy, which was applied to PubMed, EMBASE and cochrane databases. Studies investigating the diagnostic accuracy of LUS compared to thoracic CT or chest radiography (CXR) in ARDS diagnosis or focal versus non-focal subphenotypes in adult patients were included. Quality of studies was evaluated using the QUADAS-2 tool. Statistical analyses were performed using “*Mada*” in Rstudio, version 4.0.3. Sensitivity and specificity with 95% confidence interval of each separate study were summarized in a Forest plot.

**Results:**

The search resulted in 2648 unique records. After selection, 11 reports were included, involving 2075 patients and 598 ARDS cases (29%). Nine studies reported on ARDS diagnosis and two reported on focal versus non-focal ARDS subphenotypes classification. Meta-analysis showed a pooled sensitivity of 0.631 (95% CI 0.450–0.782) and pooled specificity of 0.942 (95% CI 0.856–0.978) of LUS for ARDS diagnosis. In two studies, LUS could accurately differentiate between focal versus non-focal ARDS subphenotypes. Insufficient data was available to perform a meta-analysis.

**Conclusion:**

This review confirms the hypothesis that LUS is a reliable method for diagnosing ARDS in adult patients. For the classification of focal or non-focal subphenotypes, LUS showed promising results, but more research is needed.

**Supplementary Information:**

The online version contains supplementary material available at 10.1186/s13054-024-04985-1.

## Introduction

Acute respiratory distress syndrome (ARDS) is a life-threatening respiratory condition, which manifests as acute hypoxemic respiratory failure often requiring mechanical ventilation [1, 2].

The prevalence of ARDS is high, estimated around 10% of all intensive care unit (ICU) admissions and mortality remains high [3]. Diagnosis is based on a broad set of clinical and radiological criteria lacking specificity. This results in physiological, biological, and radiological heterogeneity. The imaging criterion used in the diagnosis of ARDS is subject to considerable variability between observers and techniques. Accurate diagnosis of ARDS is of importance for the adequate management as well as clear definition of patient populations included in clinical research [3, 4].

Radiological heterogeneity in ARDS has been studied extensively and has revealed that morphological subphenotypes exist, which respond differently to ventilator treatment strategies [5]. Non-focal ARDS is defined by diffuse or patchy loss of aeration, which generally responds better to recruitment maneuvers. Focal ARDS shows predominant dorso-inferior consolidations and generally responds better to prone positioning [4]. In a prospective randomized clinical trial, a personalized treatment strategy was found to be superior only when classification was accurate and treatment was aligned with lung morphology [6], highlighting the importance of correct classification.

Since 2012, ARDS has been diagnosed by the Berlin definition [7]. In the last decade, several developments have prompted expansion of this definition. The Kigali modification is proposed for resource-constrained settings without access to CT, variable access to CXR or invasive pulse-oximetric methods for evaluating oxygenation [8]. The use of High-Flow Nasal Oxygen (HF-NO) to manage ARDS patients with severe hypoxemic patients has been investigated and the use of it exacerbated during the COVID-19 pandemic. Finally, the use of LUS is increasing rapidly in patients with acute respiratory failure. To address these changes, a global consensus was reached to update the ARDS definition [9].

This global definition acknowledges the limitations of CT or CXR for ARDS diagnosis and includes LUS as a diagnostic tool. Multiple studies have investigated LUS as a tool for identifying ARDS and differentiation between focal and non-focal subphenotypes. However, the accuracy has not yet been well defined and the approaches of LUS among studies vary. In this systematic review and meta-analysis, we aim to assess the diagnostic accuracy of LUS for diagnosing ARDS and for classification of focal versus non-focal subphenotypes.

## Objectives

### Primary objective

To evaluate the diagnostic accuracy of LUS for diagnosing ARDS in adult patients.

### Secondary objective

To evaluate the accuracy of LUS in classification of focal versus non-focal ARDS subphenotypes in adult patients.

## Method

### Study design

This is a systematic review and meta-analysis. The protocol was written according to the PRISMA-P guidelines (preferred reporting items for systematic reviews and meta-analysis protocols) and pre-registered at PROSPERO, registration number CRD42023413462.

### Inclusion criteria

We aimed to include all studies that investigated the accuracy of LUS for ARDS diagnosis compared to the Berlin definition (requiring CT or CXR) and all studies that investigated the accuracy of LUS for classification of focal versus non-focal subphenotypes compared to CT or CXR. Cohort, case–control, cross-sectional and observational studies were included. Patient population included adult patients presenting with acute respiratory failure and/or need for mechanical ventilation. The index test was LUS, all LUS protocols were included. The reference standard for ARDS diagnosis was the Berlin definition, which used CT or CXR data. The reference standard for focal versus non-focal subphenotypes was CT and/or CXR.

### Exclusion criteria

We excluded studies in pediatric patients, case reports and case series, articles published in any other language than English and studies without a reference CT or CXR. The authors made the decision to exclude non-English studies as evidence suggests that the impact on the results of systematic reviews is negligible and translation of non-English scientific papers may lead to errors in interpretation [10–13].

### Literature search

A medical librarian experienced in organizing systematic reviews was consulted to construct a robust search strategy. The search was conducted up until the 6th of February 2023, in MEDLINE via PubMed, EMBASE and Cochrane Database of Systematic Reviews. The search methods and terms used are presented in the Supplementary material.

### Selection of studies

Results were managed in an online database, Rayyan. Two independent reviewers (MB and WA) first evaluated title and abstracts. Afterwards full texts were assessed for eligibility. References of included studies were screened, and potentially eligible studies were evaluated for inclusion. Disagreement between the first two reviewers was resolved by consensus meetings with a third reviewer (PRT).

### Data extraction

Data was extracted by two independent reviewers (MB and WA). From each included study, characteristics were extracted and summarized. These included author, study design, study period, year of publication, patient characteristics, number of ARDS and non-ARDS patients, chosen LUS protocol and reference standard, primary and secondary outcomes and study limitations.

### Outcomes

The primary outcome was the accuracy of LUS in diagnosing ARDS. The secondary objective was to establish the accuracy of LUS in classification of focal versus non-focal ARDS subphenotypes. This included accuracy, sensitivity, specificity, positive predictive value, negative predictive value, positive likelihood ratio, negative likelihood ratio, diagnostic odds ratio, accuracy, AUROC and Youden’s index. Whenever a specific measure was not described in the study, raw data was used for calculation by the reviewers. Specificity, sensitivity, negative predictive value and positive predictive value were calculated by extracting raw data and implementing it in a 2 × 2 contingency table.

### Quality assessment

For quality assessment of the included studies, the QUADAS-2 tool (Quality Assessment of Diagnostic Accuracy Studies) was used by two independent reviewers (MB and WA). Disagreement was resolved by consensus meetings with a third reviewer (PRT).

### Statistical analysis and data synthesis

Pooled sensitivity and specificity estimates were obtained by applying a bivariate analysis on the raw study data, using the package “*Mada*” in Rstudio, version 4.0.3. This resulted in a summary receiver operating characteristic (sROC) curve. The sensitivity and specificity with 95% confidence interval of each separate study were summarized in a Forest plot.

For the studies that evaluated different LUS protocols or scores within the same patient cohort, the protocol that was recommended by the authors was analyzed. If no recommendation was made, the protocol with the highest accuracy was included. In the diagnostic accuracy analysis of the study of Smit et al., two different cutoff points for the LUS-ARDS score were used: a high cutoff point (LUS-ARDS score > 27) for optimal specificity and low cutoff point (LUS-ARDS score > 8) for optimal sensitivity in two different cohorts. For this meta-analysis, the sensitivity and specificity of the recommended protocol or the one with the highest accuracy was used, together with the high cutoff point for both cohorts and the low cutoff point of both cohorts from the study of Smit et al.

Furthermore, we calculated a pooled specificity and sensitivity in which we excluded studies with an ARDS incidence greater than 30%.

## Results

### Selection of studies

**Fig. 1:**
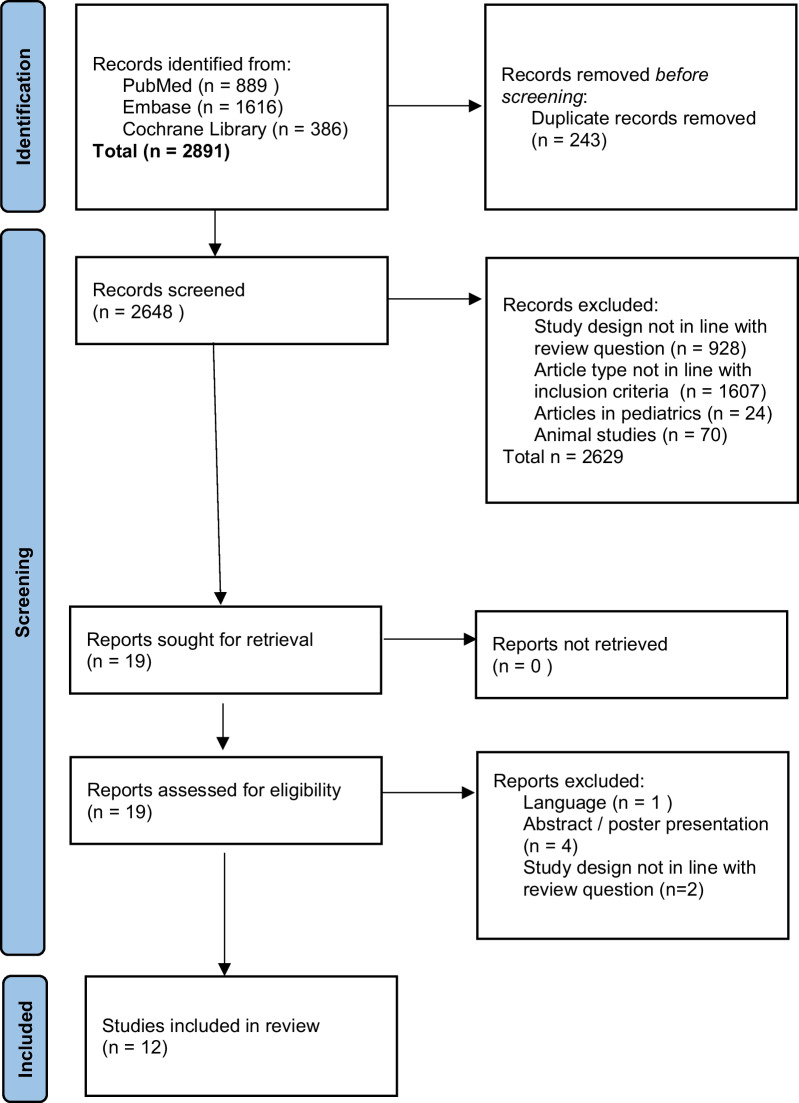
PRISMA flow diagram presenting the study search strategy and study selection process

Details regarding search strategy and study selection process are summarized in Fig. 1. After searching three databases, 2648 unique records were identified. After screening, retrieval, and assessment for eligibility, 11 studies were included in the review and underwent qualitative assessment.

### Study characteristics

Characteristics of included studies are summarized in Table 1. Most of the included studies were conducted prospectively. Two were post-hoc studies. One study retrospectively analysed results of two previously reported studies. Settings included medical, surgical, and mixed ICUs or a combination thereof. One study was conducted on a medical ward and one in the emergency department. For the index test, a variety of LUS protocols were used as shown in Table 1. None of the included studies made note of the ultrasound settings used in their LUS protocols for detecting B-lines.

**Table 1:** Study characteristics

References	Study design	Study period	Setting	Patient characteristics	N of ARDS/N patients included	Index test	Index test protocol	Reference standard	Primary outcome^a^	Secondary outcome(s)	N of operators	Study limitations
Daabis et al. [14]	Prospective	NS	Respiratory and general ICU	Acute respiratory failure	10/100	LUS	ARDS when either B-profile + PLAPS^b^ *Or* B-profile present	History	ARDS	–	1	Patients with several final diagnoses were excluded Not specified which patients received chest X-Ray and/or chest CT Criteria for ARDS diagnosis were not specified
Bass et al. [15]	Prospective	2013	General ICU	Mechanically ventilated	35/77	SpO_2_/FiO_2_ and bilateral LUS	ARDS when ≥ 3 B-lines in single frozen frame in ≥ 1 lung field	PaO_2_/FiO_2_ ≤ 300 and chest radiograph consistent with ARDS	ARDS	–	1	Repeat LUS assessments performed unblinded for medical records Sole inclusion of intubated patients Protocol using only B-lines, disregarding other LUS signs Patient repositioning avoided, reducing visibility of posterior lung fields
Huang et al. [16]	Prospective	2014–2016	General ICU	Elderly, suspected of having ARDS	33/51	12 zone LUS protocol	ARDS when bilateral lung fields with either: Interstitial syndrome Consolidation Normal or abnormal pleural line or effusion	Berlin definition	ARDS	LUS + NT-proBNP and P/F ratio for ARDS diagnosis	NS	Finite study arms Unclear interval between LUS and thoracic CT
See et al. [17]	Retrospective	2014–2017	Medical ICU	Noninvasive or invasive mechanically ventilated	216/456	Berlin LUS: Berlin definition using LUS as imaging criterium	ARDS when ≥ 1 region per hemithorax with > 2 B-lines or consolidation	Berlin definition	ARDS	Index test as predictor of ventilator free days, ICU/hospital LOS and ICU/hospital mortality	NS	Likely interobserver variability within each imaging modality Only medical ICU patients were included LUS were performed by respiratory therapists, limiting applicability
Pisani et al. [18]	Post-hoc	2016–2017	Medical and surgical ICU	Mechanically ventilated	35/152	Global and regional LUS Scores using 12 zone protocol	Lung aeration score^c^ Optimal cutoff: global lung aeration score 15	Berlin definition	ARDS	–	1	No collection of LUS features such as lung sliding, pleural line abnormalities and subpleural Consolidations, which might add to diagnostic accuracy
Pierrakos et al. [4]	Post hoc	2021/2018^c^	General ICU	Amsterdam: mechanically ventilated Lombardy: mechanically ventilated with ARDS	51/51	Global and regional LUS Scores	Lung aeration scores^c^ using Amsterdam, Lombardy and Piedmont method Anterior LUS ≥ 2 was strongly associated with non-focal ARDS	CT scan	Focal vs nonfocal lung morphology	–	NS	Validity of difference between lateral and posterior LUS scores in Amsterdam not fully assessed No COVID-19 related ARDS cases were included
Costamagna et al. [19]	Prospective	NS	General ICU	Mechanically ventilated and ARDS	47/47	Total, ventral, intermediate and dorsal LUS Scores	Lung aeration score^c^ Ventral LUS ≥ 3 strongly associated with non-focal ARDS	CT scan	Focal vs nonfocal lung morphology	–	NS	Different spatial resolution of CT and LUS may affect the evaluation of lung aeration Cohort did not include patients with Covid-19 associated ARDS
Baid et al. [20]	Prospective	2019–2021	ED	Acute respiratory failure	35/237	Anterolateral and posterior thoracic LUS using BLUE-protocol	ARDS when Subpleural anterior consolidations Reduced/absent lung sliding Thickened/fragmented pleura Inhomogeneous distributed B-lines	Berlin definition	ARDS and other chest pathologies	Time to formulate POCUS diagnosis compared to composite reference	1	Uneven distribution of differentials of acute onset dyspnoea Possible clinical bias for reference diagnosis, as ED consultants were involved in active patient management
Chaitra and Hattiholi [21]	Prospective	2017	General ICU	Acute respiratory failure	17/130	6 zone LUS using BLUE-protocol	2 ARDS criteria: (1) bilateral lung sliding + B3-lines (2) bilateral lung sliding, B3-lines + consolidation	NS	ARDS	–	1	Pleural effusion not included as distinct category ARDS and severe pneumonia not differentiated clinically Unclear diagnostic criteria for ARDS Unclear if LUS operators were blinded for clinical data
Arthur et al. [22]	Prospective	2016–2017	Medical Ward	Respiratory or cardiac reports and thoracic X-Ray performed	22/321	7 point LUS on each hemithorax (4 in sicker patients)	ARDS when Multiple B-lines, sub-pleural consolidations Air bronchograms	Final diagnosis at discharge	ARDS and other chest pathologies	Diagnostic accuracy of LUS in chest pathologies when CT scan was included in composite reference standard	1	Large frame between LUS and CXR Chance for incorporation bias, CXR included in composite reference standard Central chest and airway pathologies not seen on LUS
Smit et al. [23]	Prospective	2019–2021	General ICU	Mechanically ventilated	97/453	12-region LUS score	LUS-ARDS score 1 additional point in abnormal pleural line, consolidations, dynamic air bronchogram, pleural effusions LUS-ARDS score cutoffs: Low > 8, high > 27	Berlin definition	ARDS	LUS compared with clinical data and CXR Accuracy of LUS in scenarios where expert panel was not certain about ARDS diagnosis	3	Not all LUS signs associated with ARDS included in model Not all patients received index test because incomplete LUS regions Pragmatic sample size of validation cohort Low N nonpulmonary ARDS

Studies are presented according to publication date

For the index test, a variety of LUS protocols were used. Six studies analysed 12 lung regions, 6 per hemithorax. Three studies performed the examination according to the Bedside Lung Ultrasound Examination in Emergency (BLUE) protocol, which entails scanning 6 regions, 3 per hemithorax. One study evaluated 14 points, 7 per hemithorax. In one study, the LUS protocol was not specified. A-profile is defined as lung sliding with A-lines and < 2 B-lines per view. B-profile is defined as > 3 B-lines per view. C-profile is a consolidation

*NS* not specified, *LUS* lung ultrasound, *LOS* length of stay, *CXR* chest X-ray, *ED* emergency department, *NCIS* non-cardiogenic interstitial syndrome, *ABG* arterial blood gas

^a^Primary outcome measured diagnostic accuracy in each study included

^b^PLAPS: Posterolateral Alveolar and/or Pleural Syndrome. Presence of consolidation or effusion at the most posterior part of the lung immediately superior to the diaphragm (PLAPS point)

^c^Lung aeration score: A total of 12 lung regions is scored according to the lung profile observed per zone. Zones are given a score of 0 in A-profile; 1 in > 2 B-lines; 2 in B-lines 50% pleural line; 3 in C pattern. Global aeration score is calculated by summing the scores of all 12 lung regions, while anterior, lateral or posterior aeration scores are calculated by summing their respective lung zones

Of the nine studies investigating ARDS diagnosis, two studies used a quantitative analysis and awarded points for “A”, “B” or “C” profiles. Seven of these studies had investigators diagnose ARDS based on descriptive parameters. These parameters were multiple B-lines, pleural line abnormalities, reduced or absent lung sliding, consolidations.

The accuracy of LUS to differentiate focal from non-focal ARDS subphenotypes was the primary outcome in two studies. These two studies both applied a quantitative analysis, where points were awarded for “A”, “B” or “C” profiles and the total amount of points was added up and analysed.

### Outcomes

Eleven studies were included in total, involving a total of 2075 patients, of whom 598 (29%) had ARDS. Of these eleven studies, nine focused on LUS for ARDS diagnosis, involving 1977 patients, of whom 500 (25%) had ARDS. The two studies focusing on differentiation between focal and non-focal subphenotypes involved 98 patients, all of whom had ARDS.

1138 (55%) of the included patients were mechanically ventilated, 383 (34%) of whom had ARDS. 467 patients presented to the emergency department with acute respiratory failure, of whom 62 (13%) had ARDS.

Outcomes and diagnostic parameters of the included studies are presented in Online Appendix 1.

**Fig. 2:**
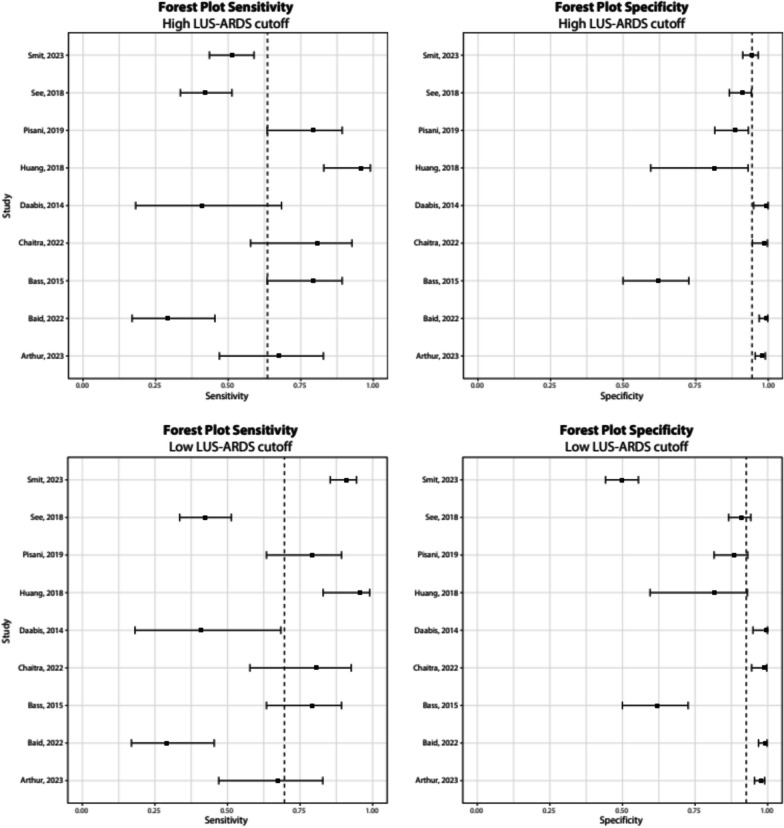
Forest plots comparing diagnostic accuracy parameters from all studies with parameters from the study of Smit et al., after using a low and high cutoff point as suggested by the authors of this study. The dashed line represents median values of sensitivity and specificity. Sensitivity and confidence intervals: Smit (high cutoff): 0.51 [0.44–0.59] Smit (low cutoff): 0.91 [0.85–0.94], Daabis: 0.41 [0.18–0.68], Bass: 0.79 [0.63–0.89], Huang: 0.96 [0.83–0.99], See: 0.42 [0.34–0.51], Pisani: 0.79 [0.63–0.89], Baid: 0.29 [0.17–0.45], Chaitra: 0.81 [0.58–0.93], Arthur: 0.67 [0.47–0.83]. Specificity and confidence intervals: Smit (high cutoff): 0.94 [0.91–0.97] Smit (low cutoff): 0.50 [0.44–0.55], Daabis: 0.99 [0.95–1.00], Bass: 0.62 [0.50–0.73], Huang: 0.82 [0.60–0.93], See: 0.91 [0.87–0.94], Pisani: 0.89 [0.82–0.93], Baid: 0.99 [0.97–1.00], Chaitra: 0.99 [0.94–1.00], Arthur: 0.98 [0.95–0.99]

**Fig. 3:**
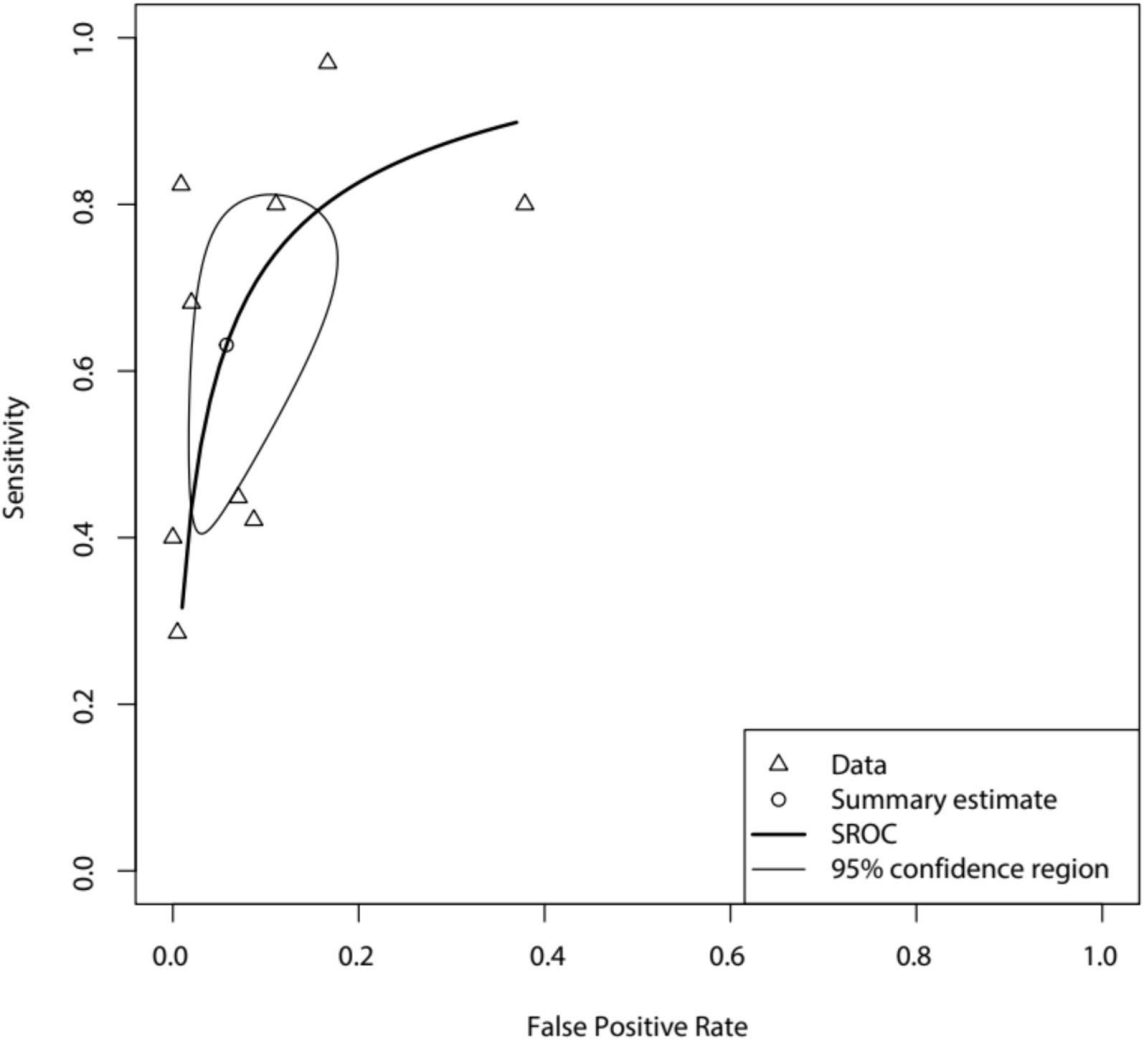
sROC curve after meta-analysis of nine studies assessing diagnostic accuracy of LUS in ARDS diagnosis

A meta-analysis comparing all studies with the diagnostic accuracy parameters using a low cutoff point from the study of Smit et al. resulted in a pooled sensitivity of 0.70 (95% CI 0.504–0.837) and pooled specificity of 0.93 (95% CI 0.789–0.977) and an area under the curve (AUC) of 0.88. Comparing the results of included studies with the high cutoff point with the study of Smit et al. resulted in a pooled sensitivity of 0.640. (95% CI 0.459–0.782) and pooled specificity of 0.94 (95% CI 0.861–0.979) and an AUC of 0.88. The Forest plots and sROC of these analyses can be found in Figs. 2 and 3, respectively.

After excluding studies with an ARDS incidence greater than 30%, a pooled sensitivity of 0.66 (95% CI 0.431–0.831) and pooled specificity of 0.96 (95% CI 0.825–0.993) and an AUC of 0.88 was found in the low cutoff group. A pooled sensitivity of 0.57 (95% CI 0.392–0.735) and pooled specificity of 0.97 (95% CI 0.930–0.990) and an AUC of 0.91 was found in the high cutoff group.

Two studies on LUS for focal versus non-focal ARDS subphenotypes also reported high specificity (71% and 100%) and sensitivity (94% and 100%).

### Risk of *bias*

Risk of bias and applicability of studies included are summarized in Table 2. In studies assessing accuracy of LUS in ARDS diagnosis, one study was scored as high risk of bias in the patient selection domain, as this study excluded patients with non-respiratory or rare causes of acute respiratory failure or patients with multiple diagnoses at the end of hospitalization. For the same reason, this study was scored as high on applicability concerns in the patient selection domain.

**Table 2:** Risk of bias and applicability assessment of included studies, following the QUADAS-2 scoring tool

Study	Risk of bias				Applicability		
	Patient selection	Index test	Reference standard	Flow and timing	Patient selection	Index test	Reference standard
*Studies comparing LUS to Berlin definition in ARDS diagnosis*							
Pisani et al. [18]	Low	Low	Low	*Unclear*	Low	Low	Low
Arthur et al. [22]	Low	*Low*	*Unclear*	Low	Low	Low	*Unclear*
Baid et al. [20]	Low	*Low*	*Unclear*	Low	Low	Low	*Unclear*
Bass et al. [15]	Low	Low	Low	Low	Low	Low	Low
Chaitra and Hattiholi [21]	Low	*Unclear*	*Unclear*	*Unclear*	Low	Low	*Unclear*
Daabis et al. [14]	*High*	Low	*Unclear*	*Unclear*	*High*	Low	*Unclear*
Huang et al. [16]	Low	Low	Low	*Unclear*	Low	Low	Low
See et al. [17]	Low	Low	Low	Low	Low	Low	Low
Smit et al. [23]	Low	Low	Low	Low	Low	Low	Low
*Studies comparing LUS to Chest CT in ARDS phenotype diagnosis*							
Pierrakos et al. [4]	Low	Low	Low	Low	Low	Low	Low
Costamagna et al. [19]	Low	Low	Low	Low	Low	Low	Low

One study was scored as unclear risk of bias as it was not specified whether researchers performing the index test were blinded for clinical data.

Four studies were scored as unclear risk of bias in the reference standard domain, as none of these studies specified the criteria used for ARDS diagnosis. For the same reason, they were scored as unclear on applicability concerns in the reference standard domain.

Four studies were scored as unclear risk of bias in the flow and timing domain, as none of these studies specified the time interval between the index test (LUS) and the diagnosis of ARDS by the reference standard.

**Fig. 4:**
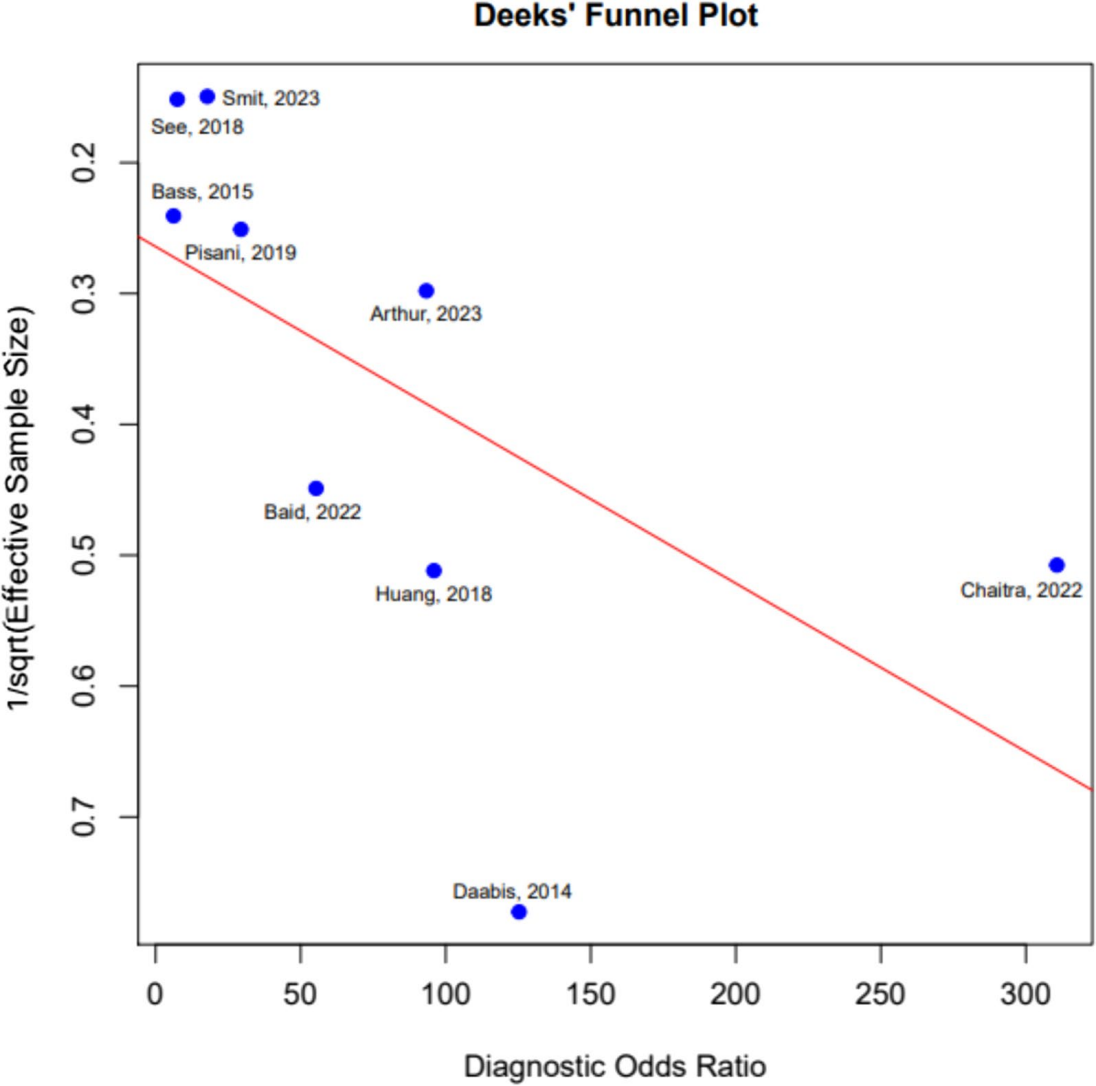
Deek’s Funnel plot for assessment of publication bias

A funnel plot was conducted to study if publication bias is present (Fig. 4).

The two studies assessing accuracy of LUS for focal versus non-focal ARDS subphenotypes diagnosis scored low on all domains of risk of bias and applicability.

## Discussion

The major findings of this systematic review and meta-analysis in adult patients on the diagnostic accuracy of LUS for ARDS compared to reference standards which included the Berlin Definition are: (1) LUS has a high pooled specificity and a moderate sensitivity in ARDS diagnosis; (2) LUS can accurately diagnose focal versus non-focal ARDS subphenotypes with a high specificity and sensitivity; (3) The prevalence of ARDS found in the included studies matches previously reported prevalence and incidence rates of ARDS in patients with respiratory failure and ICU admissions [3, 16]. This indicates generalizability of the results.

The high pooled specificity found in the current study indicates that LUS is an adequate tool for diagnosing ARDS, but less adequate in ruling out ARDS. These findings are surprising as before LUS was found to be more sensitive compared to the Berlin definition, largely due to lack of specificity of LUS criteria. This contrasting finding could be largely attributed to the specific cutoff point chosen for the meta-analysis based on cutoff points used in the included studies. To evaluate the effect of a high or low chosen cutoff point on the results, we included both the high (LUS-ARDS score > 27) and low (LUS-ARDS score > 8) cutoff points of both the cohorts in the study of Smit et al. in the meta-analysis. As shown in the results, the pooled sensitivity and specificity are almost the same, underlining the finding that LUS is a specific tool for diagnosing ARDS.

Sensitivity varied considerably between studies. An explanation for lower sensitivity could be an imperfect reference standard. Some studies only used CXR as reference standard and CXR has been shown to be an unreliable diagnostic method alone for diagnosing ARDS [24]. The difference in sensitivity between the different studies might also be attributed to the absence of a standardized LUS ARDS definition. Unclear definitions on ARDS criteria might lead to over- or under classification and impact sensitivity. Also, ARDS diagnosis might be missed by LUS if presenting with A-profile in focal ARDS subphenotype. It was hypothesized that lung ultrasound in these cases may lack sensitivity because of the posterior-predominant aspect of ARDS and these regions are more difficult to scan using LUS in critically ill patients [17], especially in less experienced operators. Furthermore, ARDS might be more difficult to detect on LUS in initial stages of the disease. In summary, LUS is an accurate tool for diagnosing ARDS and possibly less for ruling out ARDS.

To assess whether a high incidence of ARDS impacted results, we also evaluated the pooled sensitivity and specificity while in- and excluding studies with an incidence of ARDS over 30%. As can be seen in the results, this does not have a significant effect on the pooled results.

Different ARDS subphenotypes might require different treatment strategies, e.g. prone positioning for focal ARDS and lung-recruitment maneuvers for non-focal ARDS. Correct identification of ARDS subphenotype will impact treatment and outcomes in the future. This underlines the importance of accurate distinction between ARDS subphenotypes. Two studies investigated the accuracy of LUS for classification of focal versus non-focal ARDS subphenotypes compared to CT. The reported sensitivity and specificity were good [4, 18], indicating LUS as a reliable diagnostic tool for this distinction. However, since only few studies addressing this subject have been published, there is a need for further validation. In addition, a study is being conducted to see if tailoring mechanical ventilation to lung morphology assessed by LUS reduces mortality in ARDS patients compared to standard ventilation (NCT05492344).

There are many advantages of using LUS as a replacement for CT in ARDS diagnostic work-up. It can lead to an earlier start of treatment and reduce workload on ICU personnel. Furthermore, patient transport is a high-risk procedure in ICU patients and can lead to severe adverse events. LUS reduces exposure to irradiation, lowers health care costs and provides real-time examination of lung parenchyma, which also makes it ideal for follow-up of disease severity and facilitates bedside titration of ventilatory parameters. Correct subtype identification of ARDS using LUS will aid in implementing the correct treatment strategy and adequate timing of prone positioning.

There are more advantages of LUS expanding beyond ARDS diagnosis. Especially in light of the recent COVID-19 pandemic, but also in case of other respiratory emergencies, LUS can contribute to earlier identification of diseases in the emergency department (ED), allowing for earlier start of treatment and earlier admission to the ward or ICU and thus reducing the workload on ED personnel.

Of course, performing and interpreting LUS requires training. However, studies show that after 8 h of training, clinicians show proficiency in the interpretation of LUS images [25].

Currently, no consistent and/or optimal LUS protocol for diagnosing ARDS exists based on the included studies. However, among the included studies there are overlapping criteria used to establish the diagnosis. Multiple (> 2 per view) bilateral, non-homogenous B-lines, present in at least one area per hemithorax and the presence of subpleural consolidations were seen as indicative of ARDS in all studies. These criteria were confirmed by studies differentiating cardiogenic edema from non-cardiogenic interstitial syndrome, which includes ARDS but also interstitial lung disease [26–28]. Pleural line abnormalities such as irregular thickening or fragmentation were also observed, as well as areas of preserved lung parenchyma and pleural effusion. Consolidation accompanied by pleural effusion was a marker of compression atelectasis, and therefore seen as not indicative of ARDS. It seems imperative that in the future these ‘typical LUS findings’ and/or a LUS-ARDS score should be included in the new ARDS diagnostic criteria, as it was recently shown that including LUS per se increased the occurrence rate of ARDS [29].

Amongst the included studies, 6, 12 and 14 point lung ultrasound protocols were described. The optimal amount of lung regions to be included in a LUS protocol remains a subject of discussion, as 6 point protocols are more practically applicable but might lack the amount of diagnostic information compared to 12 or 18 point LUS protocols. Recent studies have shown that 6 point LUS protocols can yield similar diagnostic results compared to 12 point protocols. However, these studies have not been validated in ARDS patients [18, 30].

ARDS remains unrecognized in 20–50% of all cases, while mortality rates remain high [3]. This underlines the importance of an improved definition. Since 2012, the Berlin definition has been used as the golden standard in diagnosing patients with ARDS [7]. In 2016, the Kigali modification was proposed and validated for low-income countries, in which the bilateral opacities had to be present on either LUS or CXR [31]. Several studies already used the Kigali modification in settings with scarce access to more advanced imaging modalities [32, 33].

Also, in the global ARDS definition, lung ultrasound was proposed as an alternative for the imaging criterium for ARDS diagnosis. This review shows a good specificity but moderate sensitivity for LUS in ARDS diagnosis. LUS as a diagnostic tool for ARDS seems promising, and we support implementation of LUS in the global ARDS definition. However, we would like to highlight the importance of further prospective research and standardization of ARDS LUS definitions. In addition, an interesting recent development is the pairing of LUS with a deep learning model, which was able to distinguish COVID ARDS, non-COVID ARDS and hydrostatic pulmonary edema [34].

### Strengths and limitations

This systematic review has several strengths. To the best of our knowledge, this is the first systematic review on this subject, despite LUS already being added to the global ARDS definition. Secondly, the rigorous search strategy and method used are important strengths of this review. Another strength is the number of included studies and relatively large sample size. Of the eleven included studies, seven studies had a study population of 100 or more participants.

This review also has several limitations. Our meta-analyses included a number of small studies. Research analyzing the effect of small studies on treatment effect found that these trials often report a more positive treatment effect [35]. This might also hold true for diagnostic accuracy studies. Therefore, we advise to be careful with the interpretation of the smaller trials and limited number of cases, where a false-negative or positive results can affect accuracy more strongly than larger studies. There was sufficient data between the nine studies investigating the accuracy of LUS in ARDS diagnosis, which allowed for a meta-analysis to be performed. The studies in this review showed high sensitivity and specificity for distinguishing between focal and non-focal ARDS subphenotypes, but only two studies focused on this research question. A limited number of studies can influence the beneficial effect of the outcome researched [36]. Furthermore, there is a moderate heterogeneity between the included studies. Because there is no clear consensus yet on how to diagnose ARDS based on LUS alone, each study applied a different LUS ARDS definition, which could influence the sensitivity and specificity between different studies. A recent study showed a higher occurrence rate of ARDS when adding bilateral abnormalities as found by LUS. The authors concluded that incorporating well-defined LUS ARDS criteria in the new ARDS definitions (Kigali and Global Definition) will improve sensitivity and specificity [29]. In none of the included studies, the ultrasound preset was defined. The choice of preset can significantly influence the detection of B-lines, thereby impacting both sensitivity and specificity.

This meta-analysis assessed the risk of publication bias by funnel plot analysis. However, this should be interpreted with caution as this is primarily designed for interventional studies and it is unclear if publication bias exists for diagnostic accuracy test studies [37].

## Conclusion

This systematic review and meta-analysis demonstrate that LUS is a reliable diagnostic tool for ARDS in adult patients. The high specificity indicates that it is especially good for diagnosing ARDS, with a moderate sensitivity making it moderately reliable for ruling out ARDS. These results support the implementation of LUS in the global ARDS definition. However, given the significant heterogeneity amongst included studies, this review warrants the need for further clinical research. We would recommend a standardized LUS ARDS definition, including a preferred LUS protocol including presets for B-line recognition. This will improve homogeneity between studies and improve clinical applicability in real-life settings.

## Supplementary Information

Supplementary Material 1.

## Data Availability

All data supporting the findings of this systematic review can be found in the manuscript or supplementary material.
