# Supplementary material for: Diagnostic accuracy of lung ultrasound in diagnosis of ARDS and identification of focal or non-focal ARDS subphenotypes: a systematic review and meta-analysis
Source: Crit Care. 2024 Jul 8;28:224. doi: 10.1186/s13054-024-04985-1 (PMC11232316; doi:10.1186/s13054-024-04985-1)
Supplement: Supplementary file 1 — Supplementary Material 1. [file 13054_2024_4985_MOESM1_ESM.docx]

| **LUS vs. Composite reference standard in ARDS diagnosis** | | | | | | | | | | | |
| --- | --- | --- | --- | --- | --- | --- | --- | --- | --- | --- | --- |
| **Study** | **Protocol (if applicable)** | **Sensitivity (%)** | **Specificity (%)** | **PPV (%)** | **NPV (%)** | **LR+** | **LR-** | **DOR** | **Accuracy (%)** | **AUC** | **Youden’s index** |
| *Daabis et al 2014* | B+PLAPS-profile | 40.0 | 97.0 | 57.0 | 94.0 | 13.3* | .62* | 21.5* |  |  | .37* |
|  | B-profile | 30.0 | 97.0 | 50.0 | 93.0 | 10.0* | .72* | 13.9* |  |  | .27* |
| *Bass et al*  *2015* | 1 field | 86.0 | 38.0 | 42.0 | 83.0 | 1.4* | .37* | 3.8* | 54.5* | .73 | .24* |
|  | 2 fields | 60.0 | 77.0 | 58.0 | 79.0 | 2.6* | .52* | 5.0* | 68.3* | .73 | .37* |
|  | 3 fields | 80.0 | 62.0 | 53.0 | 85.0 | 2.1* | .32* | 6.6* | 71.3* | .73 | .42* |
| *Huang et al*  *2018* | Day 1 | 78.8 | 77.8 | 86.7* | 66.7* | 3.5* | .27* | 13.0* | 78.4* | .78 | .57* |
|  | Day 2 | 90.9 | 83.3 | 90.9* | 83.3* | 5.4* | .11* | 49.1* | 88.2* | .87 | .74* |
|  | Day 3 | 97.0 | 83.3 | 91.4* | 93.8* | 5.8* | .04* | 145* | 92.2* | .90 | .80* |
| *See et al*  *2018* | LUS1 | 69.0 | 67.0 | 66.0 | 71.0 | 2.1* | .46* | 4.6* | 68.2* |  | .36* |
|  | LUS2 | 43.0 | 83.0 | 69.0 | 62.0 | 2.5* | .69* | 3.6* | 64.0* |  | .26* |
|  | LUS3 | 22.0 | 92.0 | 72.0 | 57.0 | 2.8* | .85* | 3.3* | 59.0* |  | .14* |
|  | LUS4 | 16.0 | 95.0 | 76.0 | 56.0 | 3.2* | .88* | 3.6* | 57.7* |  | .11* |
|  | LUS5 | 9.0 | 97.0 | 75.0 | 54.0 | 3.0* | .94* | 3.2* | 55.7* |  | .06* |
|  | LUS6 | 2.0 | 99.0 | 67.0 | 53.0 | 2.0* | .99* | 2.0* | 53.1* |  | .01* |
| *Pisani et al*  *2019* | Global score | 80.0 | 88.9 | 68.3 | 93.7 | 7.2* | .22* | 32.7* | 86.8* | .91 | .69* |
| *Baid et al*  *2022* |  | 28.5 | 99.5 | 90.9 | 88.9 | 57.7* | .72* | 80.1* | 89.0* | - | .28* |
| *Chaitra et al*  *2022* |  | 82.0 | 99.0 | 93.0 | 97.0 | 82.0* | .18* | 455.6* | 95.4* | .95 | .81* |
| *Arthur et al*  *2023* |  | 68.2 | 98.0 | 71.4* | 97.7* | 34.0 | .32 | 106.3* | 96.0* | - | .66* |
| *Smit et al*  *2023* | Low cutoff (AUMC) | 87.0 | 54.0 | 46.0 | 90.0 | 1.9 | .24 | 7.9* |  |  | .41* |
|  | High cutoff (AUMC) | 55.0 | 95.0 | 84.0 | 83.0 | 11.2 | .47 | 23.8* |  |  | .50* |
|  | Low cutoff (MUMC) | 98.0 | 37.0 | 56.0 | 97.0 | 1.6 | .05 | 32* |  |  | .35* |
|  | High cutoff (MUMC) | 45.0 | 93.0 | 84.0 | 67.0 | 6.4 | .59 | 10.8* |  |  | .38* |
| **LUS vs. composite reference standard in differentiating between focal and non-focal subphenotypes** | | | | | | | | | | | |
| *Pierrakos et al*  *2021* | Piedmont | 91.3* | 75.0* | 75.0* | 91.3* | 3.6 * | .116* | 31.9* | 82.4* | .83* | .663* |
|  | Amsterdam | 91.3* | 92.9* | 92.9* | 91.3* | 12.9* | .09* | 143.3* | 92.2* | .92* | .842* |
|  | Lombardy | 100.0* | 71.4* | 74.2* | 100.0* | 3.5* | - | - | 84.3* | .86* | .714* |
| *Costamagna et al 2021* | LUS ventral score >3 | 94.0 | 100.0 | 100.0 | 86.0 | - | .05* | - | 96.0* | - | .94* |

**Appendix 1.** Results of studies diagnosing ARDS and studies differentiating between focal and non-focal ARDS

*calculated based on results given in article; AUC: Area under the curve; DOR: Diagnostic Odds Ratio; LR+: Likelihood Ratio for positive test result; LR-: Likelihood Ratio for negative test result ; NPV: negative predictive value; PPV: Positive predictive value;


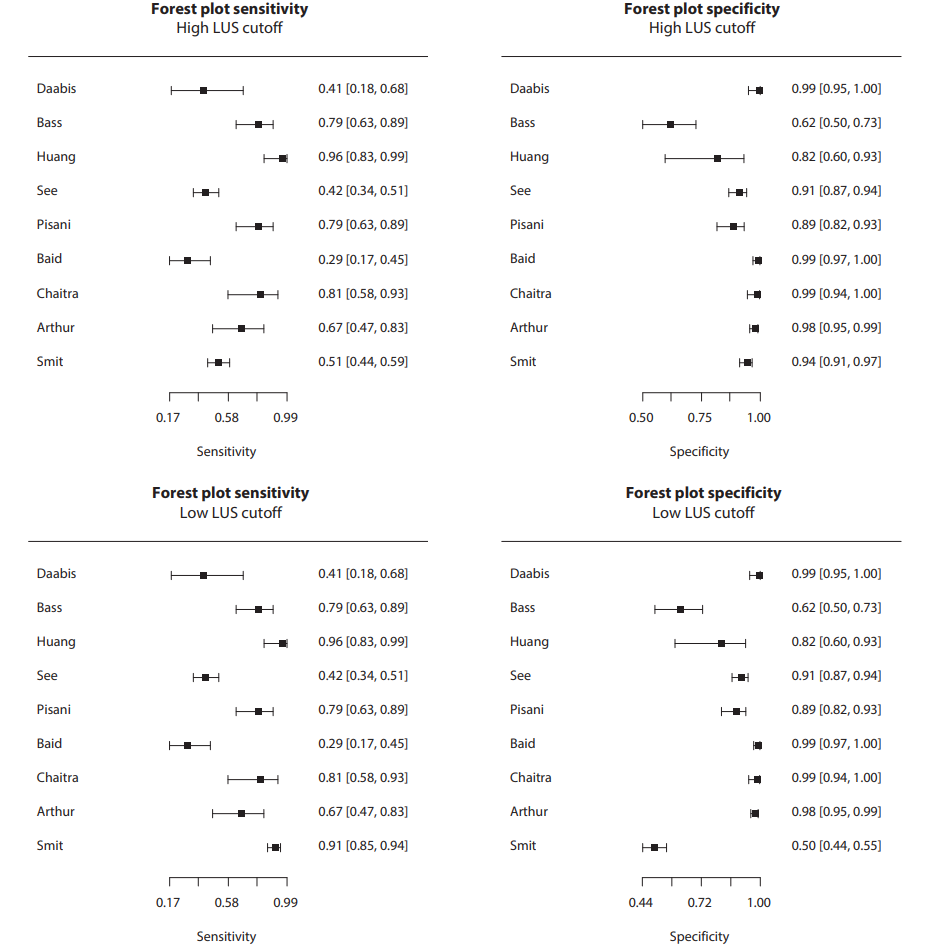


**Appendix 2**. Forest plots comparing diagnostic accuracy parameters from all studies with parameters from the study of Smit et al., after using a low and high cutoff point as suggested by the authors of this study.


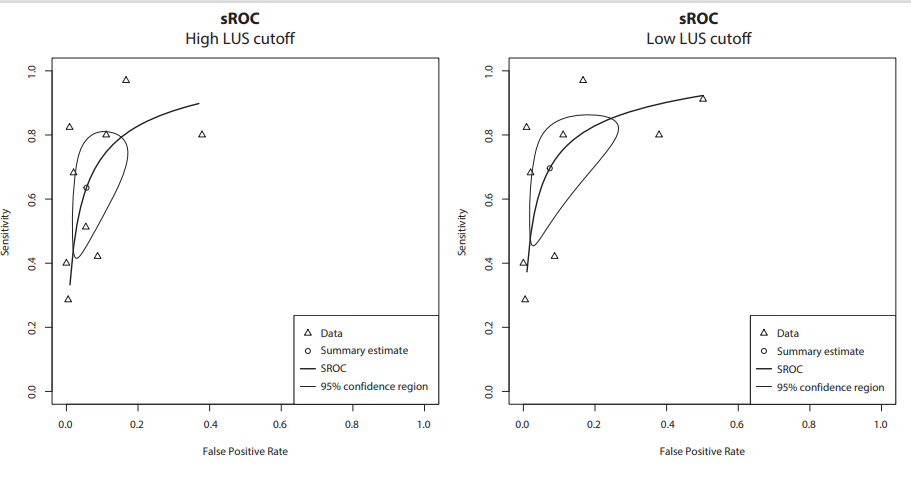


**Appendix 3**. sROC curves comparing diagnostic accuracy parameters from all studies with parameters from the study of Smit et al., after using a low and high cutoff point as suggested by the authors of this study.

**Appendix 4.** Search strategies

**Pubmed search strategy**

P

"Respiratory Distress Syndrome"[Mesh] OR Respiratory Distress Syndrome*[tiab] OR Shock Lung*[tiab] OR ARDS[tiab] OR "Acute Lung Injury"[Mesh] OR Acute Lung*[tiab]

I

"Ultrasonography"[Mesh] OR Ultrasonograph*[tiab] OR Ultrasound*[tiab] OR Echotomograph*[tiab] OR Ultrasonic*[tiab] OR Sonograph*[tiab] OR Ultrasonographic Imag*[tiab] OR Echograph*[tiab] OR Echotomograph*[tiab] OR "Ultrasonics"[Mesh] OR Ultrasonic*[tiab] OR "Ultrasonic Waves"[Mesh] OR LUS[tiab] OR LIPUS[tiab] OR "Point-of-Care Testing"[Mesh] OR Point-of-Care*[tiab] OR Point Of Care*[tiab] OR POCT[tiab] OR Bedside*[tiab] OR Bed-side*[tiab]

C

"Tomography, X-Ray Computed"[Mesh] OR Computed Tomograph*[tiab] OR Computerized Tomograph*[tiab] OR Computer Assisted Tomograph*[tiab] OR CT[tiab] OR Tomodensitometr*[tiab] OR CAT[tiab] OR Cine-CT*[tiab] OR Electron Beam Tomograph*[tiab] OR Berlin definit*[tiab] OR "Radiography"[Mesh] OR Radiograph*[tiab] OR X-Ray*[tiab] OR X Ray*[tiab] OR Roentgenograph*[tiab] OR "Diagnostic Imaging"[Mesh] OR Diagnostic Imag*[tiab] OR "X-Ray Microtomography"[Mesh] OR Microtomograph*[tiab] OR MicroCT*[tiab] OR Xray*[tiab] OR Microcomputed Tomograph*[tiab] OR Micro-computed tomograph*[tiab] OR "Radiography, Thoracic"[Mesh] OR Thoracic Radiograph*[tiab] OR Thorax radiograph*[tiab] OR Chest Radiograph*[tiab] OR "Expert Testimony"[Mesh] OR Expert Testimon*[tiab] OR Expert Witness*[tiab] OR Expert Opinion*[tiab] OR Expertis*[tiab] OR Golden stand*[tiab] OR Gold stand*[tiab]

NOT

"Respiratory Distress Syndrome, Newborn"[Mesh] OR Infantile Respiratory Distress Syndrome*[tiab] OR "Infant, Newborn"[Mesh] OR Newborn*[tiab] OR Infant*[tiab] OR Neonat*[tiab]

**Embase search strategy**

P

'respiratory distress syndrome'/exp OR (respirat*:ti,ab OR breathing:ti,ab OR lung:ti,ab OR pulmonary:ti,ab AND 'distress syndrom*':ti,ab) OR ‘Shock Lung*’:ti,ab OR ARDS:ti,ab OR ‘Acute Lung*’:ti,ab OR RDS:ti,ab

I

'echography'/exp OR Ultrasonograph*:ti,ab OR Ultrasound*:ti,ab OR Echotomograph*:ti,ab OR Ultrasonic*:ti,ab OR Sonograph*:ti,ab OR Echograph*:ti,ab OR Ultrasonic*:ti,ab OR LIPUS:ti,ab OR LUS:ti,ab OR 'point of care testing'/exp OR ('bedside':ti,ab OR 'bed-side':ti,ab AND test*:ti,ab) OR POCT:ti,ab OR (point-of-care:ti,ab OR ‘point of care’:ti,ab AND test*:ti,ab)

C

'x-ray computed tomography'/exp OR ‘x-ray computed tomography’:ti,ab OR ‘CT scan*’:ti,ab OR 'diagnostic imaging'/exp OR ‘Diagnostic Imag*’:ti,ab OR 'micro-computed tomography'/exp OR Microtomograph*:ti,ab OR MicroCT*:ti,ab OR Xray*:ti,ab OR ‘Microcomputed Tomograph*’:ti,ab OR 'micro-computed tomograph':ti,ab OR ‘thorax radiography'/exp OR ‘Thoracic Radiograph*’:ti,ab OR ‘Chest Radiograph*’:ti,ab OR 'expert witness'/exp OR ‘Expert Testimon*’:ti,ab OR ‘Expert Witness*’:ti,ab OR ‘Expert Opinion*’:ti,ab OR ‘Expertis*’:ti,ab OR ‘Golden stand*’:ti,ab OR ‘Berlin definit*’:ti,ab OR ‘gold stand*’:ti,ab OR 'radiography'/mj

NOT

'neonatal respiratory distress syndrome'/exp OR Newborn*:ti,ab OR Infant*:ti,ab OR Neonat*:ti,ab OR pediatric*:ti,ab OR neonatal:ti,ab

**Cochrane search strategy**

P

Respiratory Distress Syndrome* OR Shock Lung* OR ARDS OR Acute Lung Injur* OR Acute Lung*

OR

Respirat* OR Breath* OR Lung* OR Pulmonar*

AND

distress syndrom* OR Shock Lung* OR ARDS OR Acute Lung* OR RDS

I

Ultrasonograph* OR Ultrasound* OR Echotomograph* OR Ultrasonic* OR Sonograph* OR Echograph* OR Echotomograph* OR Ultrasonic* OR LUS OR LIPUS OR Point-of-Care* OR Point Of Care* OR POCT OR Bedside* OR Bed-side*

C

Computed Tomograph* OR Computerized Tomograph* OR Computer Assisted Tomograph* OR CT OR Tomodensitometr* OR CAT OR Cine-CT* OR Electron Beam Tomograph* OR Berlin definit* OR Radiograph* OR X-Ray* OR X Ray* OR Roentgenograph* OR Diagnostic Imag* OR Microtomograph* OR Micro-computed tomograph* OR MicroCT* OR Xray* OR Microcomputed Tomograph* OR Thoracic Radiograph* OR Thorax radiograph* OR Chest Radiograph* OR Expert Testimon* OR Expert Witness* OR Expert Opinion* OR Expertis* OR Golden stand* OR Gold stand*

NOT

Newborn* OR Infant* OR Neonat*
